# The Effectiveness of Different Teaching Modalities for the Detection of Heart Murmurs in Undergraduate Medical Education: A Review

**DOI:** 10.7759/cureus.53013

**Published:** 2024-01-26

**Authors:** Alvin Nagi, Rachel Boots, Omar Ajlouni, Sharad Nair, Abigail Werhan, Ryan Ivey, Paul Misasi

**Affiliations:** 1 Research, Kansas Health Science Center - Kansas College of Osteopathic Medicine, Wichita, USA

**Keywords:** heart auscultation, heart murmurs, undergraduate and graduate medical education, medical education, pocus (point of care ultrasound

## Abstract

One of the many physical exam skills introduced to medical students during their pre-clerkship education is cardiac auscultation, one purpose of which is to teach the detection and identification of heart murmurs. Cardiac auscultation with a stethoscope has been the standard method of teaching. Another method, point-of-care ultrasound (POCUS), has been recently introduced as another modality by which students learn to detect and identify murmurs. The emerging popularity of POCUS in undergraduate medical curricula has led many institutions to include it in their curricula; however, doing so is challenging. Not only is cost a major factor, but reorganizing curricula to allow sufficient time for POCUS training has proven to be difficult. Additionally, the presence of notable gaps in the literature regarding the efficacy of POCUS for teaching the detection and identification of heart murmur has increased scrutiny of its value. Studies that assessed teaching cardiac auscultation to medical students in their pre-clinical years via stethoscope have used different teaching methods. However, evaluation of these studies identified numerous limitations, one being little long-term retention of cardiac auscultation knowledge. Furthermore, several barriers to integration of POCUS in undergraduate medical education were identified. The purpose of this review is to synthesize the literature comparing the effectiveness of these different tools of a cardiac exam for detection of heart murmurs in undergraduate medical education and identify gaps in literature requiring future exploration.

## Introduction and background

Medical students are introduced to the fundamentals of performing physical exams during the first two years of undergraduate medical education (UME). Exam procedures and psychomotor skills are refined and solidified over the next four years as students increase proficiency and progress to clerkships and residency. It is important to have a strong foundation in these skills early in their career. Medical school faculty use different teaching methods to introduce these skills to students. One example is the auscultation of heart tones as a component of a cardiac exam for the detection and classification of heart murmurs. Heart murmurs produce qualitatively distinct sounds and sound patterns (e.g. pitch, intensity, timing in the cardiac cycle) that can be heard while auscultating different areas over the heart via a stethoscope. Cardiac auscultation with a stethoscope has been the standard method for decades for teaching medical students how to listen for normal and abnormal sounds of the heart [1]. 

Point-of-care-ultrasound (POCUS) is another modality introduced more recently in undergraduate medical education for detection of heart murmurs and is emerging as a promising technology to improve clinicians’ diagnostic accuracy [2]. POCUS is performed using a handheld ultrasound device to image the patient and has proven to be a quick, accurate, and cost-effective method that aids physicians with diagnoses [3]. It offers real-time visualization of cardiac structures and blood flow dynamics, enabling clinicians to promptly identify underlying abnormalities that contribute to murmurs. By providing immediate visual feedback at the bedside, POCUS enhances diagnostic accuracy and aids in distinguishing benign murmurs from potentially pathological ones. The overarching goal emphasizes how it can guide targeted treatment strategies, leading to improved patient outcomes [4]. For example, POCUS can help localize the origin of the murmur and characterize its intensity, timing, and location [5]. 

While the effectiveness of the different teaching methods of assessing heart sounds currently used in medical schools have been individually assessed, the effectiveness of the different methods has not been compared. A literature search was conducted via Google Scholar and PubMed. Articles on cardiac auscultation education and POCUS education were reviewed, analyzed, and classified into three categories, which will structure the current review: teaching methodology of heart murmur identification in undergraduate medical education, POCUS utilization in undergraduate medical education, and POCUS for identification of heart murmurs. Based on these findings, any proposed gaps and the need for future research were identified. 

## Review

Teaching methodology of heart murmur identification in undergraduate medical education 

Cardiac auscultation via stethoscope is the most common method of educating undergraduate medical students on how to listen to heart sounds [6]. Medical students are taught during the pre-clinical years of medical school the appropriate landmarks for auscultation on the patient during a cardiac exam. This optimizes the volume and quality of the various heart sounds to detect abnormalities in patients who may have cardiac valvular disease [6]. Students also acquire the knowledge necessary to differentiate heart murmurs by various means, which may include listening to various recordings of heart tones. Simulated patients are also employed to mimic a clinical environment, with clinical faculty present to further educate students on correct stethoscope placement. As part of the murmur identification modules in UME curricula, faculty often use descriptive medical terminology, such as “harsh systolic crescendo-decrescendo murmur,” to characterize the quality of sounds one expects to hear with various murmur types. 

Additional methods of teaching heart murmur identification include web-based content such as the Murmur Online Learning Experience (MOLE) curriculum. MOLE consists of nine online learning modules, each focusing on a common type of heart murmur and the associated sounds that accompany that murmur. In 2020, Power, Toft and Barrett studied the effectiveness of this program, which included 147 second-year medical students who were given a pretest, online learning modules, and a posttest evaluating their ability to identify murmurs. With a mean pretest score of 3.76 out of 9 and a mean posttest score of 7.14 out of 9, the authors concluded that the repetitive and descriptive nature of the online learning modules was an effective method of teaching medical students how to identify various types of heart murmurs [6]. 

However, a limitation present with this curriculum was its learning format. Because it was presented entirely online, students were not afforded the opportunity to practice the physical exam skill of cardiac auscultation. Learning how and where to effectively listen to various types of heart murmurs on real patients using a stethoscope is often a challenging transition for students because it requires being able to apply and perform the knowledge acquired from passive reading. Thus, learning modules that do not utilize the psychomotor skill of auscultating cannot promote a student’s ability to translate this online content into a clinical physical exam skill. As a result, while the MOLE does appear to be effective in teaching recognition skills, it does not ensure medical students will effectively learn how to perform the task with a stethoscope, nor can it necessarily determine the accuracy of a student in detecting a heart murmur in a clinical setting. 

Kagaya and colleagues conducted a study from 2015-2019 of 43 first-year students at Tohoku University School of Medicine, who participated in three 90-minute extra-curricular lectures on the topic of heart sounds and facilitated self-training using a cardiology patient simulator. The curriculum differed from other methods by including in-person training, self-training modules, and a cardiology patient simulator to supplement the mini-lectures. Participants were given an anonymous questionnaire at the end of the curriculum, to rate their perception of skill acquisition. Two students (5%) self-reported that they strongly agreed with the statement “I have built sufficient skills”, on a 5-point Likert scale, while 26 students (60%) agreed with that statement [7]. This suggests that a majority (65%) of first-year students were confident with their ability to detect heart murmurs via a stethoscope, and they had developed skills that better equipped them for detection of heart murmurs in clinical settings. 

Kagaya and colleagues then compared their results to those from a different study conducted in 2016-2019, which consisted of 556 fourth-year medical students who completed a three-hour mandatory attendance cardiac auscultation class. The comparison revealed that the first-year students had a statistically significant higher accuracy rate of detecting heart murmurs (85.8% vs 79.4%, p=0.001) than the fourth-year students [7]. 

These results support the interpretation that detection of heart murmurs throughout UME does not significantly improve without an effective learning method. An effective learning method allows students to acquire the knowledge necessary, while also providing the ability to apply and practice the associated skills of cardiac auscultation. Additionally, the comparison to a different study further supports the self-reported confidence levels of the participants in the Kagaya study because the higher accuracy rate aligns with their confidence levels. 

In 2013, Binka, Lewin, and Gaskin conducted a study of second-year medical students at the University of Maryland Medical Center that assessed their ability to detect 12 different heart sounds via stethoscope. The 12 heart sounds included: continuous murmur, systolic click, aortic stenosis, pericardial rub, mitral stenosis, fourth heart sound, innocent murmur, aortic regurgitation, third heart sound, tricuspid regurgitation, split in inspiration, and mitral regurgitation. Participants listened to sounds presented through a stethoscope for a period of 45 minutes and each of the 12 different heart sounds were repeated a total of six times. One hundred fifty-seven students participated in the intervention group who took a pretest before the session and a posttest immediately after the session. The intervention group demonstrated a significant increase in the ability to successfully identify eight of the 12 heart sounds. The eight heart sounds identified at higher percentages posttest were continuous murmur (81% vs 51%, p<0.001), systolic click (51% vs 22%, p<0.001), pericardial rub (94% vs 56%, p<0.001), fourth heart sound (70% vs 18%, p<0.001), innocent murmur (69% vs 37%, p<0.001), third heart sound (39% vs 6%, p<0.001), tricuspid regurgitation (62% vs 38%, p<0.001), and split in inspiration (52% vs 28%, p<0.001) [8]. These exact percentages were not provided numerically within the publication but were approximated by referencing the axes of the bar graphs depicted within the study report. 

The participants were reassessed during their third-year clerkships (i.e., one year later), the intervention group was retested on the same 12 different heart sounds. There were only three heart sounds that had a higher identification percentage when compared to the posttest; they were aortic stenosis (62% vs 35%, p<0.001), third heart sound (58% vs 38%, p<0.05), and mitral regurgitation (56% vs 40%, p<0.05) [8]. These exact percentages were not provided numerically but were approximated using the bar graphs included within the article. The control group (students who did not attend the 45-minute session) were also given the test in their third year. When compared to the control group, the intervention group had a higher percentage of correctly identified heart sounds in only one out of the 12 heart sounds, i.e., the fourth heart sound (68% vs 59%, p<0.05) [8]. Again, these exact percentages were not provided by the original authors of the study but were approximated by referencing the axes of the bar graphs included within the article. This reassessment emphasizes that the participants did not gain better diagnostic skills in a clinical setting, but were only able to gain short-term knowledge of the different sounds each murmur produces. This short-term gain of knowledge is further exhibited in the data comparing the intervention group to the control group, which had a higher percentage in one of the 12 heart sounds. 

The study authors did not offer a comparison of the retest results in the intervention group to the pretest results. Additionally, the pretest results were not compared between the intervention and control groups. Lastly, the posttest results were not compared between the intervention and control groups. This presents a significant gap in data regarding outcomes on students’ ability to identify heart sounds via auscultation. While there is some evidence that there was short-term improvement in the students’ ability to accurately detect heart sounds, there is not enough evidence to conclude that there is a long-term benefit. 

Point-of-care ultrasound utilization in undergraduate medical education 

The Accreditation Council for Graduate Medical Education (ACGME) has promoted the incorporation of POCUS training into post-graduate medical education, and it is now a required component in certain residency programs such as anesthesiology and emergency medicine [9,10]. With an increased emphasis to include ultrasound training in the graduate curriculum, a knock-on effect has occurred where ultrasound training is being integrated into undergraduate medical curriculums as well [11]. However, the integration and education of POCUS have remained a largely unexplored topic within UME. This gap in the usage and implementation of POCUS in UME may result from curricular constraints, faculty expertise, resource limitations, lack of standardization, and resistance to change [11]. 

A study conducted at Harvard Medical School in 2016 explored the integration of POCUS into their first- and second-year curriculum [11]. This study attempted to determine the feasibility and barriers of integrating a POCUS curriculum and to determine the student-perceived values and attitudes towards its inclusion. Among a cohort of 176 first-year anatomy students and 38 second-year physical diagnosis students, 94% agreed or strongly agreed with the statement that they would like to see ultrasound incorporated into the medical school curriculum [11]. Although the integration of ultrasound was largely well received by the students, the authors identified impediments to this integration, such as the substantial cost of ultrasound equipment, increased use of faculty time, and loss of curriculum time [11]. Similar conclusions regarding the significant resource and financial cost of integrating ultrasound into medical curriculum have been described by other studies as well. Deshpande and colleagues investigated the implementation of POCUS curriculum for resident training in anesthesiology and determined that such a program is quite expensive and proves to be a barrier for many residency programs that may not have the resources to support this aspect of medical training [12]. Clearly, cost and resource allocation remain large barriers to entry for POCUS education in undergraduate medical education. 

In addition to cost, another barrier of POCUS integration is the lack of standardized curriculum for POCUS education in either graduate or undergraduate medical education. A study by Cawthorn and colleagues conducted at Queen’s University School of Medicine sought to determine whether POCUS training was better delivered to pre-clinical or clinical medical students. The authors studied two groups of students: 12 first-year students and 45 third-year students. POCUS training was administered for an eight-week period by way of three different programs: Program 1 consisted of a lecture-based approach with training by a sonographer; program 2 utilized electronic education modules with training by a sonographer; and program 3 was self-directed. At the conclusion of the study, the authors noted that both first and third-year students exhibited similar improvement in the ability to interpret POCUS images with programs 1 and 2. Students in program 3 performed significantly worse in both cohorts comparatively [13]. The study concluded that student performance significantly improves with training conducted by faculty that are adequately trained in ultrasound imaging, thus providing evidence for the need to develop a standardized curriculum for POCUS education. 

The study by Cawthorn and colleagues also suggests potential advantages of delayed exposure to POCUS. The authors compared the performance of third-year students to that of the first-year students and found that the image interpretation scores were significantly higher. The results of this study suggest that the timing of exposure to cardiac ultrasound within a medical curriculum improves after the delivery of a cardiovascular system module [13]. The higher scores may be attributed to increased exposure of image interpretation and understanding of the cardiovascular system as third-year students begin clinical rotations. However, this raises the question of whether the timing of POCUS needs to be delayed for graduate training or can be taught to pre-clinical students after an organ system module has been given. A review on the topic of cardiac POCUS by Johri and colleagues in 2018 found that there were no randomized trials demonstrating that teaching cardiac POCUS at the medical student level is advantageous over teaching at the postgraduate level [14]. 

The benefits of ultrasound training within any part of an UME curriculum are still heavily contested. Feilchenfeld et al. conducted a systematic review of ultrasound in UME conducted in 2017 to parse the empirical evidence surrounding its integration, including understanding of anatomy, physical examination, and diagnostic accuracy. One of the major conclusions of this review was that POCUS integration into UME curriculum did not improve anatomical understanding, and there was inconsistent evidence of POCUS usage impacting students’ physical examination performance and diagnostic accuracy. Several of the studies the authors reviewed supported POCUS training for improving a student’s diagnostic skill set, and others blamed POCUS and other technology integrations for the decline in student physical examination skills [15]. However, these findings may be a consequence of the lack of a standardized ultrasound curriculum or poorly implemented training with ultrasound equipment. One way to better implement POCUS training within a curriculum would be to teach students to identify anatomical structures in an organ systems approach. Most medical schools today have a pre-clinical education that teaches according to organ systems. POCUS training that mimics this already implemented approach to curriculum would allow students to efficiently build connections and apply the foundational knowledge they're learning to POCUS. As such, this lack of standardization limits the generalization of findings from one setting to another. Further studies into this area are required to better elucidate the ultrasound training's effectiveness in both undergraduate and graduate medical education. 

Point-of-care ultrasound for identification of heart murmurs 

One pilot study conducted in 2021 used a prospective cohort of three, fourth-year medical students to test their ability to diagnose severe valvular dysfunction using POCUS and auscultation. Their training part of a one-week cardiology clerkship and consisted of 12 hours of handheld ultrasound operation completed in two-hour increments over the course of one month [16]. These sessions included anatomy lectures, bedside-guided lessons, and review of cases. The authors concluded that in the three students tested, POCUS was significantly better for diagnosing a combination of valvular malfunctions in the same patient when compared to auscultation. These conclusions were based on diagnostic sensitivity and specificity, calculated via SPSS version 18 software [16]. Although the small sample size in this study poses a significant limitation, these preliminary results warrant further studies that expand on these conclusions. 

To analyze the effectiveness of POCUS in a clinical setting, Draper and colleagues sought to determine the sensitivity, specificity, and predictive value for diagnosing valve disease through POCUS compared to the standard echocardiography. Their study was motivated by the desire to reduce the use of transthoracic echocardiogram (TTE), which is the standard for diagnosing asymptomatic heart murmurs [4]. While the standard is TTE, it is a lengthy exam and often requires the patient to travel to specific sites with the capability to perform the echocardiogram (i.e., personnel and equipment). The authors theorized that POCUS in the outpatient clinic setting may be an adequate substitution for TTE in patients with asymptomatic murmurs, provided comparable levels of sensitivity and specificity. The authors concluded that POCUS has a sensitivity of 88% and specificity of 95%, where auscultation had a specificity of 93% and a sensitivity of 72% [4]. The researchers concluded that in a murmur clinic, the use of POCUS as a diagnostic tool reduces demand for hospital echocardiography services [4]. As an extension to the physical exam, POCUS in this setting can provide immediate feedback for the physician and patient, while reducing time and cost of treatment. It also provides another reason to either clinically indicate a TTE or forgo it completely if it's concluded to be not needed. 

Several recent studies have demonstrated the potential for using POCUS to detect murmurs in the outpatient setting and could offer significant benefits [17]. Traditional auscultation methods often lead to subjective interpretations and diagnostic uncertainties. Diagnostic uncertainties with traditional stethoscope can be attributed to the design that involves transmission of sound proportional to frequency and a lack of sound amplification [18,19]. These issues are exacerbated in patients with larger body habitus due to thickening of the chest wall, which dampens sounds. Additionally, interpreter reliability comes into question, as there is no way to externally validate the sound data the clinician is observing during cardiac auscultation [18]. POCUS, with its real-time visualization of cardiac structures and blood flow dynamics, has the potential to provide a more definitive assessment of murmurs. It is crucial to underscore that effective application of POCUS in this context mandates proper training to ensure precise interpretation and diagnosis. In the healthcare setting this can improve the diagnostic criteria for clinically indicated tests and treatments, reduce overall diagnostic and treatment costs, and the time needed to perform these necessary steps.

## Conclusions

Despite the growing interest in incorporating POCUS into UME, there remains significant uncertainty regarding its efficacy and utility in clinical practice due to notable gaps in the literature. Auscultation via stethoscope has inherent limitations in terms of accuracy and consistency, often leading to challenges in differentiating between various murmur types. The idea that POCUS can enhance the diagnostic process by providing real-time visualization of cardiac structures and blood flow dynamics needs to be further explored. Further investigation of POCUS integration in UME curricula will help narrow the gap in the literature and provide institutions with the knowledge needed to decide whether incorporation of this diagnostic modality in their pre-clinical education is beneficial.
